# Minimal invasive brain surgery for epilepsy; can it be the future?

**DOI:** 10.1007/s00415-022-11360-z

**Published:** 2022-09-13

**Authors:** Mohamed Mustafa, Malik Zaben

**Affiliations:** 1 The Welsh Epilepsy Unit, Department of Neurology, University Hospital of Wales, Cardiff, UK; 2 Cardiff University Brain Research Imaging Centre (CUBRIC), Cardiff University, Cardiff, UK; 3 Brain Research and Intracranial Neurotherapeutics (BRAIN) Unit, Neuroscience and Mental Health Innovation Institute, Cardiff University, Cardiff, UK; 4 Department of Neurosurgery, University Hospital of Wales, Cardiff, UK

Epilepsy affects 1–2% of the general population, of whom 30–40% may be considered drug resistant. In these patients, surgery is increasingly considered as the treatment of choice. However, conventional epilepsy surgery is invasive and associated with significant risks and overall intervention rates remain low. However, minimally invasive surgery, such as stereotactic radiosurgery and MRI-guided laser interstitial thermal surgery, have become popular alternative treatments, especially when a patient is not fit for a major open surgical procedure.

In this month’s journal club, we will discuss three papers addressing these two modalities used in minimally invasive epilepsy surgery. The first is a clinical trial which randomizes patients with pharmacoresistant temporal lobe epilepsy to either stereotactic radiosurgery or open temporal lobectomy. The second is a systemic review and meta-analysis assessing outcomes of MRI-guided laser interstitial thermal therapy in the treatment of temporal epilepsies, extratemporal epilepsies and hypothalamic hamartoma. The last paper is a retrospective study comparing outcomes of MRI-guided laser interstitial thermal therapy and open temporal lobectomy.

## Radiosurgery versus open surgery for mesial temporal lobe epilepsy: the randomized, controlled ROSE trial

Open surgery for intractable temporal lobe epilepsy results in a seizure freedom rate of 60–90% but remains major and frightening step for some patients. Stereotactic radiosurgery is a non-invasive which can target deep and otherwise difficult-to-access lesions. It delivers a focal dose of high-energy radiation using three-dimensional image guidance. It usually requires a short post-procedure hospital stay, but may also be done as an out-patient procedure.

In this multi-centre single-blinded trial, 58 patients were recruited; 31 patients in the stereotactic surgery (SRS) arm and 27 patients in the anterior temporal lobectomy arm (ATL). Seizure remission was achieved in 52% and 78% of patients who underwent SRS and ATL, respectively. The result did not prove non-inferiority of SRS compared with ATL. The pattern of seizure remission was apparent immediately following the procedure in the ATL arm, but in the SRS arm maximal remission of 74% occurred 34–36 months post intervention. There were 14 adverse events in 12 (39%) in the SRS arm, of which 5 were considered serious (seizure exacerbation, headache, cerebral edema, and new neurological deficit) and 9 non-serious. In the ATL arm there were five adverse events in three (11%) patients (two serious (wound scalp dehiscence, and cerebritis) and three non-serious). All adverse events in the SRS arm occurred within 11 months and in the ATL group side effects were confined to 3 months of surgery.

**Comment:** This study supports SRS as a safe treatment option for patients with drug-resistant epilepsy. Although ATL was more effective in achieving seizure freedom with fewer adverse events, SRS appears to be a viable alternative treatment for those considered inappropriate for open surgery. A major limitation was the sample size, likely compounded by difficulty recruiting as well as the relatively brief duration of follow-up. Although achieving timely seizure freedom for patients with epilepsy is clearly important, the efficacy of SRS appears to increase with time and may have improved further with longer duration of follow-up. Finally, the majority of patients in the ATL arm received intravenous steroids as part of the intervention protocol, which may have contributed to the differences observed in adverse event frequency, particularly immediately following intervention.

*Barbaro NM et al. (2018) Epilepsia 59(6):1198–1207.*

## Surgical outcomes between temporal, extratemporal epilepsies and hypothalamic hamartoma: systematic review and meta-analysis of MRI-guided laser interstitial thermal therapy for drug-resistant epilepsy

MRI-guided laser interstitial thermal therapy (MRgLITT) is a minimally invasive technique that ablate epileptic foci using laser energy. The technique may be particularly useful for those patients who have deep epileptic foci and/or those who would not tolerate open surgery. In the absence of a well-constructed randomized controlled clinical trial this systemic review summarizes current evidence.

Literature was systemically reviewed utilizing PubMed, MEDLINE, and EMBASE databases from 2012 to 2020. The Engel Epilepsy Surgery Outcome Scale (Freedom from disabling seizures; class 1, rare disabling seizures; class 2, worthwhile improvement; class 3, no worthwhile improvement; class 4), which reports seizure freedom was used as the primary endpoint. Twenty-eight studies incorporating 559 patients were included. Hypothalamic hamartomas had the highest seizure freedom rate (class 1) of 67%. Mesial temporal lobe epilepsy and extratemporal epilepsy have a similar seizure freedom rate (class 1) of 56% and 50% respectively. The incidence of adverse events was similar between all groups (19%). The most frequent adverse event was visual field deficit (*n* = 30) which occurred predominantly in patients with mesial temporal lobe epilepsy (*n* = 22). This was followed by intracranial hemorrhage in 13 (2.5%). Re-operation rate was 9%. Supplementary data also demonstrated that MRgLITT was beneficial in most patients with MRI-defined lesions and that the rate of seizure freedom declined over time.

**Comment:** This study offers encouraging data on the safety and efficacy of MRgLITT. Heterogeneity between studies was an important limitation including discrepancy of ablation volume. In addition, despite MRgLITT being a relatively novel surgical technique, the authors excluded studies published only as abstracts and published case series with < 5 patients. Post treatment adverse events were mainly transient, although no data were presented on the frequency and nature of more permanent deficits. Finally, most of the studies included were performed within a limited geographic area, which may limit generalization, and it is clear that an International multi-center randomized trial comparing the efficacy of MRgLITT and open ATL is urgently needed.

*Barot, Niravkumar* *,* *et al. (2022) Journal of Neurology, Neurosurgery & Psychiatry 93.2: 133–143.*

## Inverse national trends of laser interstitial thermal therapy and open surgical procedures for refractory epilepsy: a Nationwide Inpatient Sample-based propensity score matching analysis

In this study, the United States’ National Inpatient Sample (NIS) database was used to identify the practice, pattern and outcome of laser interstitial thermal therapy (LITT) and open surgery for patients with refractory epilepsy. The data included records between 2012 and 2016 and identified 7045 patients who underwent epilepsy surgery (400 LITT (5.7%), 6645 open surgery (94.3%)). Median length of hospital stay post-procedure was 1 day for LITT, and 4 days for open surgery (*p* < 0.0001). Adverse effects were more frequent in patients undergoing open surgery compared to LITT (15% vs 4%). Post procedure, 96% of patients treated with LITT were discharged home compared to 86% of patients undergoing open surgery. Median index hospitalization charges were significantly higher in open surgery ($124,012) compared to LITT ($108,332) (*p* < 0.0001). A propensity score matching technique was used to match individuals in both groups, but did not significantly change results although did abolish the difference observed in incidence of post-surgical complications.

**Comment:** This study faced multiple challenges in data collection including lesion size as well as location and size of ablation. In addition, there was no data offered for out-patient follow-up, which could have provided useful additional information about an individual’s wider clinical condition following surgery. The relatively small sample size for patients with LITT is a further limitation. Furthermore, despite the intensive resources and technology required for LITT, it has the potential to reduce overall costs, mainly by decreasing length of hospital stay, which may also reduce complications resulting from prolonged inpatient hospital stays.

*Sharma, M et al. (2020). Neurosurgical Focus FOC, 48(4), E11.*

